# Selective Changes of Resting-State Brain Oscillations in aMCI: An fMRI Study Using ALFF

**DOI:** 10.1155/2014/920902

**Published:** 2014-04-14

**Authors:** Zhilian Zhao, Jie Lu, Xiuqin Jia, Wang Chao, Ying Han, Jianping Jia, Kuncheng Li

**Affiliations:** ^1^Department of Radiology, Xuanwu Hospital of Capital Medical University, Beijing 100053, China; ^2^Beijing Key Laboratory of Magnetic Resonance Imaging and Brain Informatics, Beijing 100053, China; ^3^Department of Neurology, Xuanwu Hospital of Capital Medical University, Beijing 100053, China

## Abstract

Mild cognitive impairment (MCI) refers to a transitional state between normal aging and dementia and is a syndrome with cognitive decline greater than expected for an individual's age and educational level. As a subtype of MCI, amnestic mild cognitive impairment (aMCI) most often leads to Alzheimer's disease. This study aims to elucidate the altered brain activation in patients with aMCI using resting-state functional magnetic resonance. We observed Frequency-dependent changes in the amplitude of low-frequency fluctuations in aMCI patients (*n* = 20), and normal subjects (*n* = 18). At the same time, we took gray matter volume as a covariate. We found that aMCI patients had decreased amplitude of low-frequency fluctuation signal in left superior temporal gyrus, right middle temporal gyrus, right inferior parietal lobe, and right postcentral gyrus compared to the control group. Specially, aMCI patients showed increased signal in left superior and middle frontal gyrus. Our results suggested that increased activation in frontal lobe of aMCI patients may indicate effective recruitment of compensatory brain resources. This finding and interpretation may lead to the better understanding of cognitive changes of aMCI.

## 1. Introduction

The concept of mild cognitive impairment (MCI) refers to subjects who experience cognitive impairments but who are not demented [1]. MCI is a syndrome with cognitive decline greater than expected for an individual's age and educational level but not interfering notably with activities of daily living. The prevalence of MCI is about 15% in adults older than 65 years and more than half of MCI patients progress to dementia within 5 years. The common outcome of nonamnestic MCI is frontotemporal dementia or dementia with Lewy bodies. Patients with the amnestic subtype of MCI (aMCI) have an annual conversion rate of 6–25% to Alzheimer's disease (AD). As such, aMCI has been regarded as a prodromal stage of AD [2–6]. Over the past decade, significant progress has been accomplished in our understanding of its epidemiology, risk factors, natural history, and treatment. Although there remain some controversies surrounding MCI, it is increasingly recognized that MCI should be handled as a clinically defined condition. Because the standard diagnostic procedure of aMCI primarily relies on neuropsychological examinations, there is strong demand to develop neuroimaging techniques as reliable surrogate MCI markers. Whereas structural MRI provides important diagnostic and prognostic information, fMRI remains promising as an imaging marker of MCI, including aMCI.

Recently, low-frequency fluctuations (LFF) fMRI has gained increased attention based on observations using fMRI approaches and direct current coupled electroencephalographic scalp recordings [7–9]. Spatially organized and temporally coherent fluctuations in the low-frequency range (0.01–0.1 Hz) have been at the center of attention, as the BOLD signal displays a spatial structure similar to task function-related activation [10–12].

Most studies of resting-state functional magnetic resonance imaging (fMRI) have applied the temporal correlation in the time courses to study the functional connectivity between different brain regions. Biswal and coworkers have shown that spontaneous low-frequency (<0.08 Hz) fluctuation (LFF) is highly synchronous among motor cortices [10]. Recently, resting-state synchronization has also been investigated in patients [13–16] and in healthy subjects [17–19]. The power of (LFF) may also be used as a biomarker to assess cerebral spontaneous activity [20]. ALFF is defined as the total power within the frequency range between 0.01 and 0.1 Hz. Our study aims to evaluate the ALFF signal in reflecting cerebral physiological states in aMCI patients and healthy subjects. We evaluate whether the ALFF abnormalities in aMCI have similar distribution pattern as independent component analysis (ICA) approach [21]. Several studies had shown grey matter loss in aMCI or MCI [22–25] and regional brain atrophy may lead to artificial reduction in low-frequency fluctuation [26]. In order to directly test our hypothesis and improve the statistical strength, we took gray matter volume as a covariate.

## 2. Materials and Methods

### 2.1. Study Population

Thirty-eight right-handed subjects were recruited. Participants were divided into two groups based on their clinical profiles: twenty participants were classified as aMCI patients and the other eighteen as healthy controls. Patients were recruited from a memory clinic at the Department of Neurology and healthy controls were recruited from a community investigation of epidemiological research. Informed consent was approved by the Medical Research Ethics Committee of Xuanwu Hospital and obtained from all subjects. Prior to resting-state fMRI scanning, examination of each subject included medical history, neurological examination, informant interview, neuropsychological assessment 4 including mini-mental state examination (MMSE), clinical dementia rating (CDR), activity of daily living scale, Hachinski ischemic scale, Hamilton rating scale for depression, auditory verbal learning test (AVLT), structural MRI, and standard laboratory tests.

aMCI diagnosis was established according to the criteria for amnestic MCI [5, 6]. To be diagnosed as having MCI, patients had to fulfill the following criteria: (a) impaired memory performance on a normalized objective verbal memory test, (b) recent history of symptomatic worsening in memory, (c) normal or near-normal performance on global cognitive tests [mini-mental state examination (MMSE) score > 24] as well as on activities of daily living scale, (d) global rating of 0.5 on the CDR scale, with a score of at least 0.5 on the memory domain, and (e) absence of dementia. Stroke, psychiatric diseases, drug abuse, moderate to serious hypertension, and systematic diseases were ruled out. Memory complaints or neurological deficiencies were not observed in the healthy controls with normal conventional brain MR imaging and an MMSE score ≥ 28. Demographics and neuropsychological findings of aMCI patients and healthy elderly are shown in Table 1. Demographics of aMCI patients and healthy controls, including age, sex, and education years, were matched between the two groups. The age of participants was equally distributed between the two diagnostic groups (*t* = 2.06, *P* = 0.57, two-sample two-tailed *t*-test) with similar medians and ranges. However, the groups were significantly different with regard to MMSE scores and AVLT scores (*P* < 0.001, two-sample two-tailed *t*-test).

### 2.2. Data Acquisition

MRI data were collected on a 3T scanner (Siemens, Trio, Erlangen, Germany), with an eight-channel receiver coil. Subjects were instructed to keep their eyes closed and to refrain from initiating goal-directed, attention-demanding activity during the scanning sessions, and resting-state fMRI was acquired. fMRI was acquired using gradient echo planner imaging (EPI) for a 6 min and 20 s period, resulting in a total of 124 volumes (repetition time (TR)/echo time (TE) = 3000/30 ms, flip angle = 90°, field of view (FOV) = 256 × 256 mm^2^, matrix size = 64 × 64, 28 slices, slice thickness = 4 mm, and 0 mm interslice gap). A T_1_WI anatomical dataset was obtained from each subject using a magnetization-prepared rapid acquisition gradient echo sequence (TR/TE = 1900/2.2 ms, inversion time (TI) = 900 ms, flip angle = 9°, FOV = 256 × 256 mm^2^, matrix size = 224 × 256, 176 slices, voxel size = 1 × 1 × 1 mm^3^). According to the inclusion criteria, T_2_WI and FLAIR scans were reviewed to exclude the presence of remarkable macroscopic brain abnormalities.

### 2.3. Voxel-Based Morphometry Data Processing

Structural MRI data analysis was performed using an optimized VBM protocol (http://dbm.neuro.uni-jena.de/vbm/) under SPM5 (http://www.fil.ion.ucl.ac.uk/spm/), which included slice timing, motion correction, spatial normalization, and smoothing.

Images were segmented into grey matter, white matter, and cerebral spinal fluid (CSF) [27]. The images were then normalized to the Montreal Neurological Institute (MNI) template, and then the parameters were applied to normalize individual T_1_ images separately. The fully normalized images were once again segmented into grey matter, white matter, and CSF. The normalized and modulated white matter and gray matter images were smoothed using a 4 mm × 4 mm × 4 mm full-width half-maximum (FWHM) Gaussian kernel for subsequent statistical analysis. Then, the grey matter volume of the aMCI patients and the normal controls was calculated. The result was also entered into the following statistical analysis to examine the effects of GM atrophy on the functional results.

### 2.4. Resting-State fMRI Data Processing and Statistics

Functional MRI data were processed using the statistical parametric mapping (SPM5, http://www.fil.ion.ucl.ac.uk/spm/), which included slice timing, motion correction, and spatial normalization. ALFF analysis was performed by using resting-state fMRI data analysis toolkit (http://resting-fmri.sourceforge.net/). The procedure for calculating the ALFF is similar to the previous studies [20, 28]. The filtered time series was transformed to a frequency domain with a fast Fourier transform (FFT) (parameters: taper percent = 0, FFT length = shortest) and the power spectrum was then obtained. The square root was thus calculated at each frequency of the power spectrum and the averaged square root was obtained across 0.01–0.08 Hz at each voxel. This averaged square root was taken as the ALFF. For standardization purposes, the ALFF of each voxel was divided by the global mean ALFF value within a brain mask, which was obtained from the intersection of the brain of all subjects' T_1_ images. The analysis included the grey matter as a covariate. A two-sample *t*-test was performed to test the ALFF difference between aMCI patients and normal controls. Voxels with a corrected *P* value < 0.01 (single voxel threshold of *P* < 0.05 and cluster size 540 mm^3^, using the AlphaSim program with parameters: FWHM = 8 mm, with mask) were considered significant.

## 3. Results

### 3.1. Demography and Neuropsychological Test

Demographic characteristics and neuropsychological scores were shown in Table 1. There were no significant differences between the two groups in gender, age, and years of education, but the MMSE and AVLT (auditory verbal learning test) scores were significantly different (*P* < 0.05) between the two groups.

### 3.2. Brain Regions of Decreased Gray Matter Volume between the Two Groups

**Figure 1:**
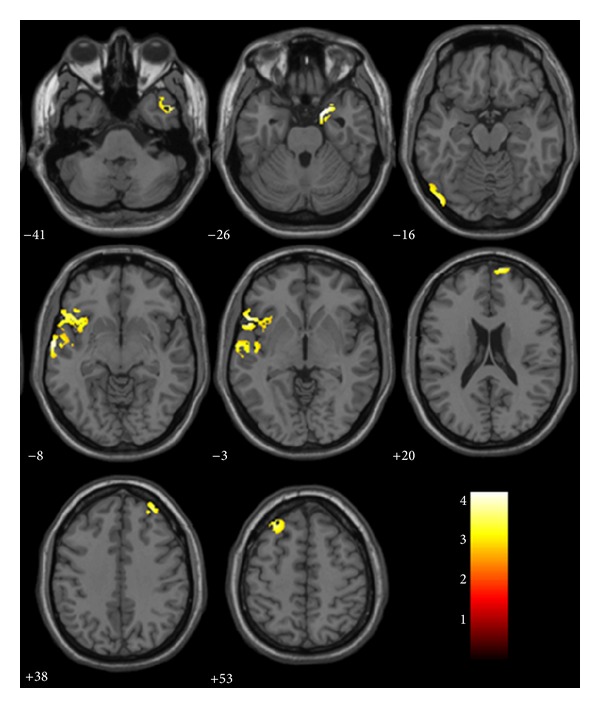
VBM analysis showed brain regions of significant reduction of gray matter volume in aMCI patients relative to controls on axial position images. Reader's right is subjects' right.

**Figure 2:**
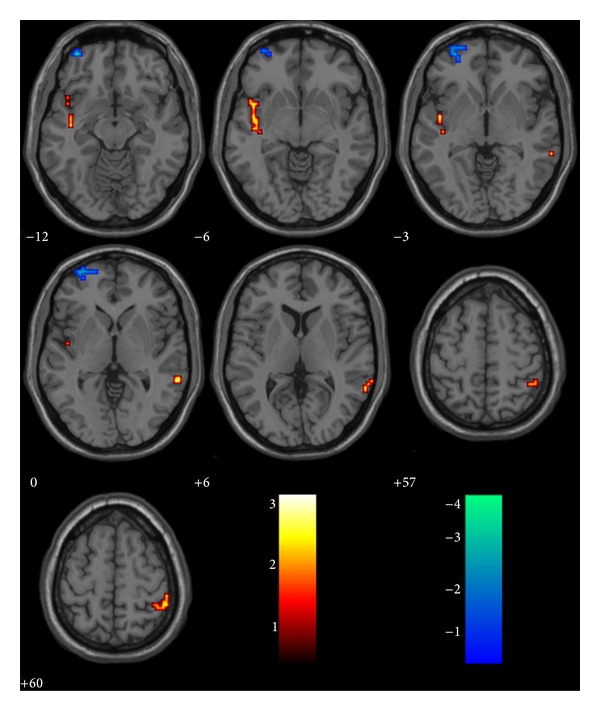
Decreased and increased activity in aMCI patients shown in axial projection compared to the healthy elder. Hot: NC-aMCI (decreased); winter: aMCI-NC (increased).

To assess possible causes of reduced functional activity, we analyzed our data for structural differences between both study groups. The aMCI patients showed widespread reduction in gray matter volume in the right uncus, the bilateral inferior, superior, and middle frontal gyrus, the bilateral medial temporal gyrus, the left inferior temporal gyrus, the left superior temporal gyrus, the right superior parietal gyrus, and the left middle occipital gyrus (*P* < 0.001, corrected) (Figures 1 and 2, Table 2). Some of these areas did overlap with those regions found to be altered in the patient group using ALFF.

### 3.3. Whole Brain Functional Alteration Data: The Between-Group Differences

Figure 2 shows the statistical map resulting from comparison of the changes of ALFF in different brain areas in healthy elderly versus aMCI patients. ALFF was higher in controls than patients in left superior temporal gyrus, right middle temporal gyrus, right inferior parietal lobe, and right postcentral gyrus. ALFF was significantly higher in patients than controls in left superior, middle frontal gyrus (see Figure 2 and Table 3).

## 4. Discussion

In the current study, we reported abnormal ALFF in aMCI patients compared to healthy controls. ALFF was higher in controls than patients in the left superior temporal gyrus, right middle temporal gyrus, right inferior parietal lobe, and right postcentral gyrus. Patients had higher ALFF than controls in left superior, middle frontal gyrus. Thus, our data suggested that there are abnormalities in LFOs in aMCI patients. The current findings add to a literature suggesting abnormalities of neural synchrony in aMCI and extend these findings to the LFO domain.

The reduced LFO in the IPL and the temporal gyrus is consistent with previous studies of aMCI. More recently, functional imaging studies have suggested that memory processes are subserved by a set of distributed, large-scale neural networks. Specific regions of the default network are selectively vulnerable to early amyloid deposition in AD. The lateral parietal and temporoparietal areas are involved in the default network [29]. Using ICA, [21] found that right inferior parietal lobule exhibited decreased functional activity in aMCI compared to normal control, and [30] found reduced activity in the patient group in bilateral superior parietal lobes. Previous studies have demonstrated that some regions constitute a structurally and functionally connected neuronal network that supports the default function of the human brain. The IPL is one of the brain regions that constitute the major posterior extent of the default mode network (DMN). Reduced right IPL activity indicated impaired memory functional system in aMCI patients. Episodic memory function is severely affected in AD and is also the key early marker for prodromal stages such as MCI [31]. This may suggest that the aMCI is prodromal stage of AD.

An interesting finding of the present study is that, in aMCI without motor clinical impairment, LFOs abnormalities occur also in the motor system, mainly in right postcentral gyrus. The abnormal change in motor system may resemble those described for the cognitive network. Recent evidence indicates that part of classical motor areas may have nonmotor cognitive functions in addition to the well-known motor functions [32]. It is well known that the parietal cortex, which has extensive connections with regions of the frontal lobes, where it sends rich sensory information for movement control, is involved in the elaboration of somatosensory inputs and in movement preparation and planning [33]. These results may suggest an overactivation of selected areas of the sensorimotor network. However, there may be other explanations that we have not known. More work needs to be done to study the role of the postcentral gyrus in the aMCI patients.

The areas of increased amplitude in patients are mainly located in left frontal regions previously associated with abnormal function in this disorder. The frontal cortices are key regions involved in human memory processing [21, 34]. This is consistent with the assumption that AD and MCI patients may be able to use additional neural resources in prefrontal regions to compensate for losses in cognitive function [21, 35, 36]. Critically, activity in this network of regions was correlated with the ability of the patients to perform the tasks accurately. Patients who had more activity in bilateral prefrontal areas were better able to perform tasks of semantic and episodic memory [35, 37]. However, it still remains unclear whether these alterations may be compensatory to maintain memory performance in the setting of early AD pathology or instead represent evidence of excitotoxicity and impending neuronal failure.

There were several limitations in the current study that need to be addressed. First, the education years among the aMCI subjects we selected were large, between 0 (illiteracy) and 19; this may have confounding effects on the results; in future studies we would group our subjects across their education with more aMCI patients. Second, the subjects were instructed to keep their eyes closed during the resting scan; subjects may have looked at something subconsciously and we did not obtain such behavioral data, or some subjects sleep during the examination. Previous studies have suggested that the alpha power is related to different resting conditions 13 [38, 39]. Future studies would benefit from the use of eye tracking or visual monitoring equipment during the resting-state session.

## 5. Conclusions

In conclusion, we have demonstrated ALFF differences in aMCI patients using functional MRI method. This resting-state fMRI study suggests that the abnormal spontaneous activity of these regions may indicate the underlying pathophysiology of aMCI. Research is ongoing to determine if these early alterations will serve as sensitive predictors of clinical decline and, eventually, as markers of aMCI progress to AD.

## Figures and Tables

**Table 1:** Demographics and neuropsychological findings of aMCI patients and healthy elderly.

	aMCI patient (*n* = 20)	Healthy elderly (*n* = 18)
Age	65.11 ± 9.92	66.81 ± 7.43
Female/male	12/8	10/8
Education (y)	11.84 ± 3.32	12.02 ± 2.93
MMSE	25.21 ± 2.24	29.31 ± 1.22
AVLT, learning A1	7.22 ± 1.73	3.95 ± 1.82
AVLT, learning A2	9.39 ± 2.73	5.15 ± 1.42
AVLT, learning A3	10.94 ± 2.15	6.05 ± 2.54
AVLT, delayed recall A4	11.33 ± 2.52	4.75 ± 3.51
AVLT, recognition A5	13.06 ± 1.80	7.80 ± 3.53
CDR	0.5	0

**Table 2:** Areas of gray matter loss in aMCI patients compared with healthy controls.

	Region	BA	Voxels	MNI			*T*-score
				*x*	*y*	*z*	
NC-MCI	Left MTG	35	23	−18	−27	−24	5.26
	Right MTG	30	14	15	−33	−12	4.05
	Left MOG	34	12	−9	−3	−24	3.93
	Right MFG	37	12	60	−45	−9	3.79
	Right uncus	44	12	63	6	21	3.75
	Left IFG	10	10	−30	51	−3	3.32
	Right SFG	22	14	60	6	3	3.26

*MTG: medial temporal gyrus; MOG: middle occipital gyrus; MFG: middle frontal gyrus; IFG: inferior frontal gyrus; SFG: superior frontal gyrus.

**Table 3:** Resting-state activities in controls and aMCI patients (amplitude of low-frequency fluctuations).

Condition	Connected regions	BA	Cluster	*t*-score	Coordinates (MNI)		
NC-MCI	Left superior temporal gyrus	22	37	3.10	−45	−3	−3
				2.69	−45	6	−6
	Right middle temporal gyrus	21	16	2.85	60	−42	0
				2.67	63	−48	6
	Right inferior parietal lobe	40	16	2.577	51	−42	60
	Right postcentral gyrus	20		2.303	51	−33	60
	Right inferior parietal lobe	40		2.131	42	−45	57
MCI-NC	Left middle frontal gyrus	10	28	4.148	−36	57	−12
	Left superior frontal gyrus	11		3.577	−30	57	−3
				2.43	−24	63	−3
